# Age-dependent interactions of maxillary sutures during RME and their effects on palatal morphology: CBCT and dental cast analysis

**DOI:** 10.1007/s00056-022-00429-z

**Published:** 2022-10-07

**Authors:** Gero Stefan Michael Kinzinger, Jan Hourfar, Charlotte Buschhoff, Frank Heller, Heike Maria Korbmacher-Steiner, Jörg Alexander Lisson

**Affiliations:** 1 Department of Orthodontics, Saarland University, Kirrberger Straße 100, 66424 Homburg/Saar, Germany; 2 Praxis für Gesichtschirurgie und Implantologie, Krefeld und Viersen, Germany; 3 Department of Orthodontics, University Dental Hospital Marburg, Marburg, Germany

**Keywords:** Biomechanics, Median palatal suture, Transverse palatal suture, Cone-beam computed tomography (CBCT), Dental cast analysis, Biomechanik, Sutura palatina mediana, Sutura palatina transversa, Digitale Volumentomographie (DVT), Modellanalyse

## Abstract

**Purpose:**

The effects of rapid maxillary expansion (RME) on the transverse palatine and midfacial sutures have been extensively scrutinized. Unlike the dentition stage, age-dependency was not yet regarded when investigating morphological changes of the tooth-bearing palate. Therefore, the first aim of the present study was to analyse age-dependent sutural and morphological changes of the palate in selected patients by cone-beam computed tomography (CBCT) and dental cast analysis. Secondly, age-dependent effects of RME on width, height, and depth of the palate in the region of the maxillary palatine processes were investigated by a comprehensive dental cast study, so that the combination of results could be used to provide a biomechanical explanation of the occurring changes.

**Methods:**

CBCT datasets of 9 patients (between 7.3 and 13.8 years) were measured around the median palatal suture and compared with the results of an individualised dental cast analysis. In addition, possible effects on other maxillary sutures were investigated. In the dental cast study, changes after RME in the tooth-bearing palate were analysed three-dimensionally in 60 children and adolescents. It was possible to divide those into three equally sized, age-dependant groups (PG1: < 10 years, *n* = 20; PG2: ≥ 10 < 12 years, *n* = 20; PG3: ≥ 12 years, *n* = 20).

**Results:**

The CBCT analysis reveals age-related differences in sutural responses. The opening width of the median palatine suture decreases cranially (frontal) and dorsally (horizontal). The opening mode thus changes from parallel to triangular in both planes. The transverse palatine suture completely opens in younger patients only (PG1 and PG2). The width increases are always significant in all patients. While in PG1 the width increase is greater posteriorly than anteriorly, this is always reversed in PG2 and PG3. The palatal height always increases significantly anteriorly, but posteriorly only in the youngest patients (PG 1) median and paramedian. In PG 2 and PG 3, the posterior height change is very small. That is the reason why the anteroposterior comparison reveals a much more pronounced height increase anteriorly than posteriorly.

**Conclusion:**

The comparison of selected CBCT data with a dental cast analysis allows the conclusion that the maxillary expansion after RME in children up to 10 years is rather parallel, whereas it occurs V‑shaped (anterior > posterior transversal, inferior > superior vertical) with increasing age, especially in adolescents from the age of 12. In addition to an age-progressive rigidity of the pterygopalatomaxillary junction, morphological changes of the transverse palatine suture during growth seem to be causal. Thus, age-dependent effects of palatal expansion occur due to a positional change of maxillary centres of rotation and resistance. From dental cast measurements, especially at the skeletal–basal level, conclusions can be drawn about the median palatal suture opening mode.

## Abstract (de)

**Ziel:**

Die Effekte der „rapid maxillary expansion“ (RME) auf die Sutura palatina mediana und Suturen des Mittelgesichts wurden umfangreich wissenschaftlich evaluiert. Morphologische Veränderungen des zahntragenden Gaumens dagegen sind bislang nur in Abhängigkeit vom Dentitionsstadium, nicht aber vom Alter der Patienten untersucht worden. Ziel der vorliegenden Studie war deshalb zunächst, an selektierten Patienten unterschiedlicher Altersstufen durch DVT(digitale Volumentomographie)- und Modellanalyse altersabhängige suturale und morphologische Veränderungen des Gaumens zu analysieren. Anschließend sollten altersabhängige Auswirkungen der RME auf Breite, Höhe und Tiefe des Gaumens im Bereich der Processus palatini der Maxilla durch eine umfangreiche Modellstudie bestimmt werden, sodass die Kombination der Ergebnisse abschließend zur biomechanischen Erklärung der auftretenden Veränderungen genutzt werden konnte.

**Methoden:**

Bei 9 Patienten (zwischen 7,3 und 13,8 Jahren) wurden DVT-Datensätze im Bereich der medianen Gaumensutur vermessen und mit den Ergebnissen einer individualisierten Modellanalyse abgeglichen. Zusätzlich wurden mögliche Auswirkungen auf weitere maxilläre Suturen untersucht. In der Modellstudie wurden bei *n* = 60 Kindern und Jugendlichen Veränderungen des zahntragenden Gaumens nach RME dreidimensional analysiert. Es konnte dabei in 3 gleich große Patientengruppen in Abhängigkeit vom Alter unterteilt werden (PG1: < 10 Jahre, *n* = 20; PG2: ≥ 10 < 12 Jahre, *n* = 20; PG3: ≥ 12 Jahre, *n* = 20).

**Ergebnisse:**

Die DVT-Analyse offenbart altersabhängige Unterschiede der suturalen Reaktionen. Die Öffnungsweite der Sutura palatina mediana reduziert sich in der Vertikalen besonders nach kranial, in der Transversalen nach dorsal. Infolgedessen ändert sich der Öffnungsmodus in beiden Ebenen von parallel zu triangulär. Die Sutura palatina transversa ist nur bei jüngeren Patienten (PG1 und PG2) durchgehend geöffnet. Die durch Modellanalyse ermittelten Breitenzunahmen sind bei allen Patienten immer signifikant. Während bei PG 1 die Breitenzunahme im posterioren Bereich größer ist als anterior, verhält sich dies bei PG 2 und PG 3 umgekehrt. Bei älteren Patienten ist die Breitenzunahme auf allen Ebenen anterior deutlich größer als posterior. Die Gaumenhöhe nimmt anterior bei allen Patienten signifikant zu, im posterioren Bereich dagegen median sowie rechts und links paramedian nur bei den jüngsten Patienten (PG 1). Bei PG 2 und PG 3 ist die Veränderung der Höhe posterior sehr gering. Deshalb ist hier im anterioposterioren Vergleich die Höhenzunahme anterior wesentlich deutlicher ausgeprägt als posterior.

**Schlussfolgerung:**

Der Vergleich selektierter DVT-Daten mit den Ergebnissen der Modellvermessung lässt die Interpretation zu, dass die maxilläre Erweiterung nach RME bei Kindern bis zum 10. Lebensjahr eher gleichförmig, mit zunehmendem Alter – besonders bei Jugendlichen ab dem 12. Lebensjahr – dagegen V‑förmig (anterior > posterior transversal, inferior > superior vertikal) erfolgt. Ursächlich dafür scheinen neben einer altersprogredienten Rigidität der pterygopalatomaxillären Verbindung insbesondere morphologische Veränderungen der Sutura palatina transversa im Wachstumsverlauf zu sein. Somit beruhen altersabhängige Effekte der Gaumennahterweiterung auf einer sich verändernden Lage maxillärer Rotations- und Widerstandszentren. Aus der Modellvermessung, besonders auf skelettal-basaler Ebene, lassen sich Rückschlüsse auf die Art der medianen Gaumensuturöffnung in der Transversalebene ziehen.

## Introduction

During rapid maxillary expansion (RME, sometimes called rapid palatal expansion [RPE]), the palatine process of the maxilla and the horizontal laminae of the palatine bone are separated by application of force in children and adolescents, while the pterygoid processes in the caudal region spreads laterally [39]. These combined effects lead to the therapeutically desired basal expansion in patients with pronounced maxillary constriction.

In adults, supportive surgery is necessary for successful RME treatment. This is thought to be caused by an age-related increase in the sutural bone density of the median palatal suture, reduced bone elasticity and fusion of the circummaxillary sutures during skeletal maturation [1, 11, 25, 30, 36]. Without pterygopalatomaxillary separation during surgically supported RME, only an anteriorly open triangular expansion can be achieved, whereas additional pterygopalatomaxillary separation can realise a parallel expansion [30]. Surgical separation of the maxilla from the sphenoid is also recommended as a preventive measure to avoid tension-related complications in the region of the skull base [18].

In children and adolescents, there is no unanimous opinion in the literature on when conventional RME treatment leads to which expansion mode. Many studies focused on the transverse plane and here especially on changes in the median palatal suture. However, in their systematic reviews, both Bazargani et al. [4] and Liu et al. [27] found primarily no consensus on whether RME treatment leads to triangular, i.e. anteriorly greater expansion, or to parallel expansion of the median palatal suture. However, patient age, measurement methods and recording techniques vary greatly in these studies.

The morphological changes of the tooth-bearing palate are of particular interest for orthodontic treatment. Kinzinger et al. [23] could demonstrate with a dental cast analysis for the first time that the therapeutic effects of rapid maxillary expansion on palatal morphology vary in patients with two different dentition stages. They concluded that RME should be performed in the early mixed dentition if parallel palatal expansion is desired. In later dentition stages, however, maxillary expansion occurs triangularly.

Increasing obliteration tendency or sutural bone density of the median palatal suture cannot be the sole cause of such different morphological treatment reactions of the tooth-bearing palate. Rather, an additional interaction with transversely running palatal sutures is to be suspected, which causes an age-dependent change in the position of rotation and resistance centres.

In the present study, the results of a three-dimensional (3D) radiological diagnosis are first compared with a limited cast analysis. Then, for the first time, the therapeutic effects of RME treatment on the morphology of the tooth-bearing palate are retrospectively quantified three-dimensionally with a larger number of patients subdivided according to chronological age using study casts. The combination of results is used to highlight and discuss the respective influence of the transverse palatine suture and the pterygopalatomaxillary junction [41] on the morphological changes.

## Study aims

The goals of the present study were to investigate the following questions:

- Do visual inspection and metric analyses of cone-beam computed tomography (CBCT) datasets provide information about the cause of age-dependant changes after RME?
- Can conclusions be drawn about possible different opening modes of the median palatal suture and interactions with other structures, especially the transverse palatal suture?
- Do width, height and depth of the tooth-bearing palate show age-dependant differences anteriorly and posteriorly after RME?
- Can different sutural responses be the cause of different therapeutic effects?
- Are age-related changes in the position of centres of resistance and rotation conceivable?

## Materials and methods

### Patients

Of 83 patients treated between 2016 and 2022 by the same practitioner with a dentally anchored RME appliance with hyrax screw for forced maxillary expansion, 60 (36 female, 24 male) patients were included in the study. The inclusion criteria were no previous orthodontic treatment, Caucasian origin, pronounced maxillary arch constriction, unilateral or bilateral crossbite, and the presence of two high-quality dental casts each (T1 = insertion of the RME and T2 = immediately after removal of the RME), and nearly identical wearing time and number of hyrax screw activations.

CBCT datasets were obtained from 9 patients from the collective 1 to 2 weeks after the last activation of the hyrax screw (CBCT Carestream CS 8100 3D appliance, Carestream Dental LLC, Atlanta, GA, USA). The indication for this was the possible risk of root resorption of the lateral incisors or first premolars due to their proximity to the upper canines. CBCT scans prior to treatment were not available. It can be assumed that there was no influence upon relevant sutures prior to therapy in any of the patients.

The 60 patients were divided into three groups of 20 patients each according to the chronological age at T1: Patients up to 10 years were assigned to group 1 (PG1), from 10–12 years to group 2 (PG2), and from 12 years to group 3 (PG3). The youngest patient was 7.28 years old at treatment begin, the oldest patient 16.45 years. The average age was 11.33 ± 2.60 years (PG1: 8.57 ± 0.81 years, PG2: 10.94 ± 0.63 years, PG3: 14.43 ± 1.41 years). The RME appliance remained in situ for an average of 6.13 ± 1.50 months (PG1: 6.06 ± 1.69 months, PG2: 6.26 ± 1.25 months, PG3: 6.07 ± 1.60 months). The wearing time and hyrax screw activations were recorded for each group (mean 25.15 ± 5.37, PG1: 25.10 ± 6.4; PG2: 25.20 ± 5.0, PG3: 25.15 ± 4.83) to ensure evaluation of treatment effects independently of both factors.

## RME appliance

An RME appliance with exclusively dental anchorage was used in all patients in the present study to ensure comparability. This appliance with a hyrax screw (palatal screw type S, Forestadent, Pforzheim, Germany with a lift height of 0.2 mm) was fixed with two prefabricated bands on the maxillary first molars and fitted with two occlusal rests on the first premolars or deciduous molars, respectively. The appliance was activated twice daily until the desired transverse arch expansion including a moderate overcorrection was achieved. The appliance then remained passively in situ for about 6 months.

## Metric analysis and visual assessment of the CBCT datasets

**Fig. 1:**
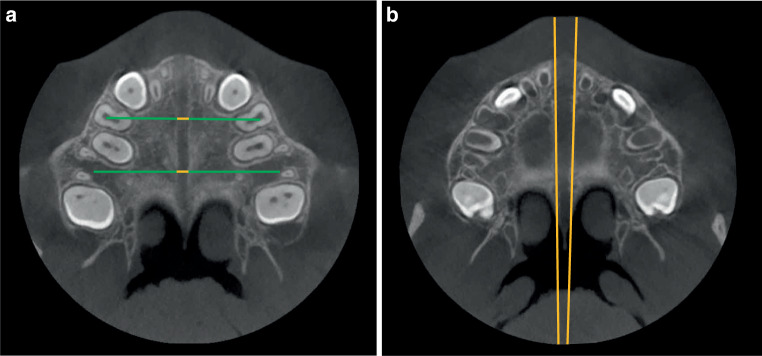
**a**,**b** Cone-beam computed tomography image analysis of the median palatal suture in the transverse plane. The opening width of the suture was measured at the level of the Pont measurements (**a** mm: *yellow sections*) and the expansion angle was determined (**b** degrees) **a**,**b** DVT(digitale Volumentomographie)-Bildanalyse der Sutura palatina mediana in der Transversalebene. Die Öffnungsweite der Sutur wurde auf Höhe der Pont-Messpunkte gemessen (**a** mm: *gelbe Abschnitte*), und der Expansionswinkel wurde bestimmt (**b** Grad)

The following skeletal reference points, distances and angles were examined on the CBCT datasets of 9 patients (*n* = 3 per group):

In the transverse plane, the opening width of the median palatal suture was measured at projected connecting distances in the region of the anchorage teeth (Fig. 1a), the transverse expansion angle (Fig. 1b) was determined, and the anterior–posterior ratio was calculated.

**Fig. 2:**
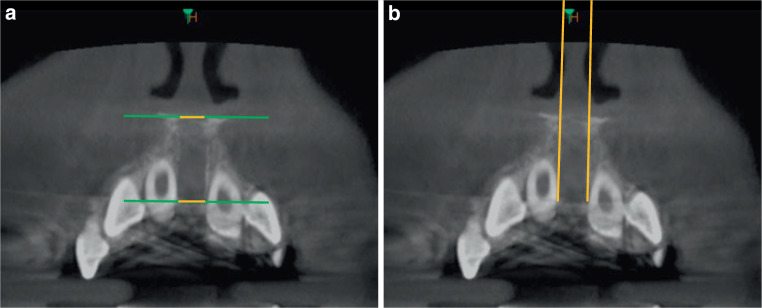
Cone-beam computed tomography analysis of the median palatal suture in the frontal plane. In the region of the opened suture, the opening width of the suture was measured caudally and cranially (**a** mm: *yellow sections*) and the angle of expansion (**b** degrees) was determined DVT(digitale Volumentomographie)-Analyse der Sutura palatina mediana in der Frontalebene. Im Bereich der geöffneten Sutur wurde deren Öffnungsweite kaudal und kranial gemessen (**a** mm: *gelbe Abschnitte*), und der Expansionswinkel (**b** Grad) wurde bestimmt

In the frontal plane, the superior and inferior distances of the open intermaxillary suture were measured (Fig. 2a), their ratio (inferior/superior) calculated, and the frontal expansion angle determined (Fig. 2b).

Finally, a 3D reconstruction was made from the CBCT datasets for visual assessment of the surrounding sutures (intermaxillary, median palatine, transverse palatine and pterygopalatomaxillary sutures).

## Dental cast analysis

A total of 120 dental casts were measured, taken before insertion of the RME appliance (T1) and immediately after its removal (T2). The dental casts were digitised for the analysis.

**Fig. 3:**
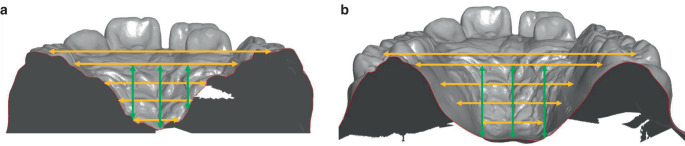
Dental cast analysis anteriorly (**a**) and posteriorly (**b**) at the level of the Pont measurements: Measurements of dental arch and jaw widths (mm, *yellow*) and measurement of palatal height (mm, *green*) to determine the ratio of width anterior/posterior (a/p) and the ratio of height a/p Modellanalyse anterior (**a**) und posterior (**b**) auf Höhe der Pont-Messpunkte: Messung der Zahnbogen- und Kieferbreiten (mm, *gelb*) und Messung der Gaumenhöhe (mm, *grün*) zur Bestimmung des Verhältnisses der Breite a/p und des Verhältnisses der Höhe a/p

The dental arch width was measured anteriorly at the premolars or deciduous molars and posteriorly at the first permanent molars according to Pont [34]. The width of the palate was measured between the most coronal points of the gingival margin at the first premolars or deciduous molars and the first permanent molars (gingival–alveolar plane). Starting from this plane, the width was determined in 2 mm steps ascending cranially up to 6 mm (skeletal–basal plane; Fig. 3). On these three exemplary vertical planes (dental, gingival–alveolar and skeletal–basal, i.e. gingival–alveolar +6 mm cranially), the ratio of the anterior–posterior width was determined to record the quality of the transverse expansion (values < 1 = inverse V‑shaped/delta-shaped; 0 = parallel, > 1 = V-shaped/triangular).

The palate height was determined by measuring the perpendicular junction of the raphe median line with the junction lines of the most coronal points of the gingival margin at the first premolars or deciduous molars and the first permanent molars. Anteriorly and posteriorly, the palatal height was measured median and 5 mm right (first quadrant) and left (second quadrant) paramedian of the palatal centre (Fig. 3). The ratio of anterior–posterior height was calculated median and paramedian (values < 1 = relatively greater increase in posterior height; 0 = equal change in anterior and posterior height, > 1 = relatively greater increase in anterior height).

The width ratio and the height ratio indicate changes in the palatal depth in the sagittal plane.

## Comparison of CBCT and dental cast analysis

The measurements of 9 patients with CBCT scans were correlated with the results of the cast analysis on the three defined levels (dental, gingival–alveolar and skeletal–basal) and their anterior–posterior ratio to allow a direct comparison between the results of the width measurements on the dental cast and the RME effects upon the median palatal suture.

## Statistical method, error of the method

Normal distribution was evaluated visually and with the Shapiro–Wilk test after data collection. Treatment-associated changes in variables were analysed for intragroup comparisons using the linked t‑test. Differences between the groups were assessed using analysis of variance (ANOVA). Post hoc testing was performed using the Tukey test. Homogeneity of variance was confirmed using the Levene test. Mean and standard deviation (SD) as well as the confidence interval (CI) were reported for each variable. Statistical significance was assumed at *p*-values < 0.05. The significance level was defined as follows: *p* ≥ 0.05 not significant, *p* < 0.05 significant, *p* < 0.01 highly significant, *p* < 0.001 highly significant. Of the dental casts, 25% were randomly selected and measured again by the same investigator after 3 months to determine the combined method error (MF) according to Dahlberg [9]. The error of the method for linear (height, width) and angular measurements was calculated with the formula MF = √(∑d^2^/2n), with *d* as the difference between two measurement results and *n* as the number of repeated measurements. The MF in the present study was < 1 for all measurements (height 0.61 mm, width 0.55 mm).

## Results

### CBCT scan evaluation

Visual evaluation of the sutures in the CBCT scans showed that the median palatal suture/intermaxillary suture was open in all 9 patients. It can be assumed that this was a therapeutic consequence of RME treatment. Opening of the transverse palatal suture can be detected only in patients from PG1 and PG2. On the contrary, it appears to be partially or completely closed in patients from PG3. The paired pterygopalatomaxillary sutures were only partially open in the youngest, 7.3-year-old patient, they were otherwise closed without exception. Only clearly open sutures in the median plane were measured. Due to the limited comparability of width measurements with different hyrax screw activation, it should be noted that absolute values are less meaningful than ratio values in the patients (Table 1).

**Table 1:** Patients with a cone-beam computed tomography (CBCT) scan: patient group, age, gender, mean rapid maxillary expansion (RME) wear time, number of hyrax activations, visual inspection, and CBCT measurements at T2 (mm/°), cast measurement ∆T2 − T1 (mm) Patienten der DVT(digitale Volumentomographie)-Studie: Patientengruppe, Alter, Geschlecht, Tragedauer der RME („rapid maxillary expansion“), Anzahl der Hyraxschraubenaktivierungen, visuelle Befundung und metrische Analyse der DVT-Aufnahmen zum Zeitpunkt T2 (mm, Grad), Modellanalyse ∆T2 − T1 (mm)

Patient no.	1	2	3	4	5	6	7	8	9
*Patient group (PG)*	PG 1	PG 1	PG 1	PG 2	PG 2	PG 2	PG 3	PG 3	PG 3
*Age (years)*	7.3	8.2	9.5	10.1	10.2	10.8	13.3	13.5	13.8
*Gender (m/f)*	f	m	f	m	m	f	m	m	m
*Wear time RME (months)*	5.2	6.0	5.0	6.0	5.1	5.2	5.3	5.0	5.4
*Hyrax activation*	30	26	23	26	30	20	20	22	26
*Visual suture inspection (open/partially open/closed)*									
– Intermaxillary suture	Open	Open	Open	Open	Open	Open	Open	Open	Open
– Median palatal suture	Open	Open	Open	Open	Open	Open	Open	Open	Open
– Transverse palatal suture	Open	Open	Open	Open	Open	Open	Part.o.	Clsd	Clsd
– Pterygopalatomaxillary suture right	Part.o.	Clsd	Clsd	Clsd	Clsd	Clsd	Clsd	Clsd	Clsd
– Pterygopalatomaxillary suture left	Part.o	Clsd	Clsd	Clsd	Clsd	Clsd	Clsd	Clsd	Clsd
*Intermaxillary suture —frontal plane*									
– Width (mm)									
Superior	4.0	4.6	3.2	2.9	3.2	2.8	2.5	2.4	2.3
Inferior	4.2	4.7	3.4	3.1	3.8	3.2	3.4	3.8	3.7
– Ratio									
Inferior/superior	1.05	1.02	1.06	1.07	1.19	1.14	1.36	1.58	1.61
– Frontal expansion (°)	1.0	1.0	2.0	2.0	2.5	3.0	3.5	4.0	4.5
*Median palatal suture—transverse plane*									
– Width (mm)									
IV-IV/4‑4	3.8	3.1	2.6	2.7	2.8	2.5	2.8	2.6	2.9
6‑6	3.7	3.3	2.3	2.2	2.4	2.1	2.2	1.9	1.9
– Ratio a/p									
IV-IV/4-4/6‑6	1.03	0.94	1.13	1.23	1.17	1.19	1.27	1.37	1.53
– Transverse expansion (°)	1.5	1.5	2.0	2.5	2.5	2.5	3.0	3.5	4.0
*Cast measurements (mm)—transverse plane*									
– Width anterior (4-4)									
Dental	6.2	6.0	5.5	4.9	5.1	3.9	3.5	4.1	4.7
Gingival–alveolar	5.4	5.9	5.3	5.1	4.0	4.0	3.2	3.8	3.9
Skeletal–basal	2.1	2.3	2.5	2.5	2.8	2.0	2.2	3.1	3.2
– Width posterior (6-6)									
Dental	6.2	6.3	5.3	4.2	4.6	3.5	2.8	3.0	3.1
Gingival–alveolar	4.7	6.1	4.8	4.4	3.6	3.5	2.4	2.7	2.5
Skeletal–basal	2.0	2.5	2.2	2.0	2.5	1.7	1.8	2.2	2.0
– Ratio a/p									
Dental	1.00	0.95	1.04	1.17	1.11	1.11	1.25	1.37	1.52
Gingival–alveolar	1.15	0.96	1.10	1.16	1.11	1.14	1.33	1.41	1.56
Skeletal–basal	1.05	0.92	1.14	1.25	1.12	1.18	1.22	1.41	1.60

*a/p* anterior/posterior, *Part.o.* partially open, *Clsd* closed

**Fig. 4:**
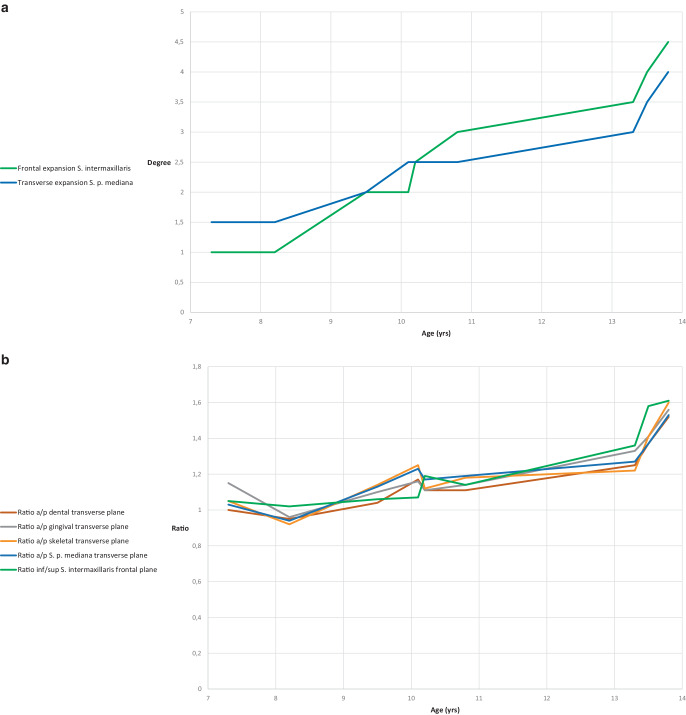
Graphs showing angle of expansion (**a**) and inferior/superior (inf/sup) and anterior/posterior (a/p) ratios (**b**) between 7 and 14 years. The individual values were determined by cone beam computed tomography (CBCT) measurements, calculated on the defined dental, gingival and skeletal transverse planes on the dental cast measurements, and plotted as a function of patient age between 7 and 14 years Graphische Darstellung des Expansionswinkels (**a**) und des Verhältnisses inferior/superior und anterior/posterior (**b**) zwischen 7 und 14 Jahren. Die einzelnen Werte wurden durch DVT(digitale Volumentomographie)-Messungen ermittelt, auf den definierten dentalen, gingivalen und skelettalen Transversalebenen an den Modellen berechnet und als Funktion des Patientenalters zwischen 7 und 14 Jahren aufgetragen

In the frontal and transverse planes, the same tendency can be observed with increasing patient age. In the frontal plane, the intermaxillary suture initially shows a wide, almost parallel opening in the youngest patients. With increasing age, the extent of the opening decreases both superiorly and inferiorly. As this is more pronounced superiorly than inferiorly, both the ratio and the frontal expansion angle change with age and present a clear development towards a V-shaped sutural opening (Fig. 4a).

The measurement of the median palatal suture in the transverse plane shows almost equal values at the measurement sections in the datasets of the youngest patients and indicates an almost parallel expansion, while a decreasing opening of the suture occurs from anterior to posterior with increasing age, thus producing a V-shaped opening. The age-dependent different expansion modes of the suture were confirmed by the anteroposterior ratio: with values around 1, a parallel opening of the suture could be detected for the younger patients and a V-shaped opening with increasing age. The values of the transverse expansion angle confirm this trend. In the transverse plane, too, the extent of expansion decreases overall, here more posteriorly than anteriorly (Fig. 4a).

In the transverse plane, the individual comparison of the dental cast ratio data with the sutural expansion shows rather similar width changes for each of the 9 patients; this applies particularly to the skeletal–basal measurement planes (Fig. 4b).

## Comparison of three patients between PG1, PG2 and PG3

**Fig. 5:**
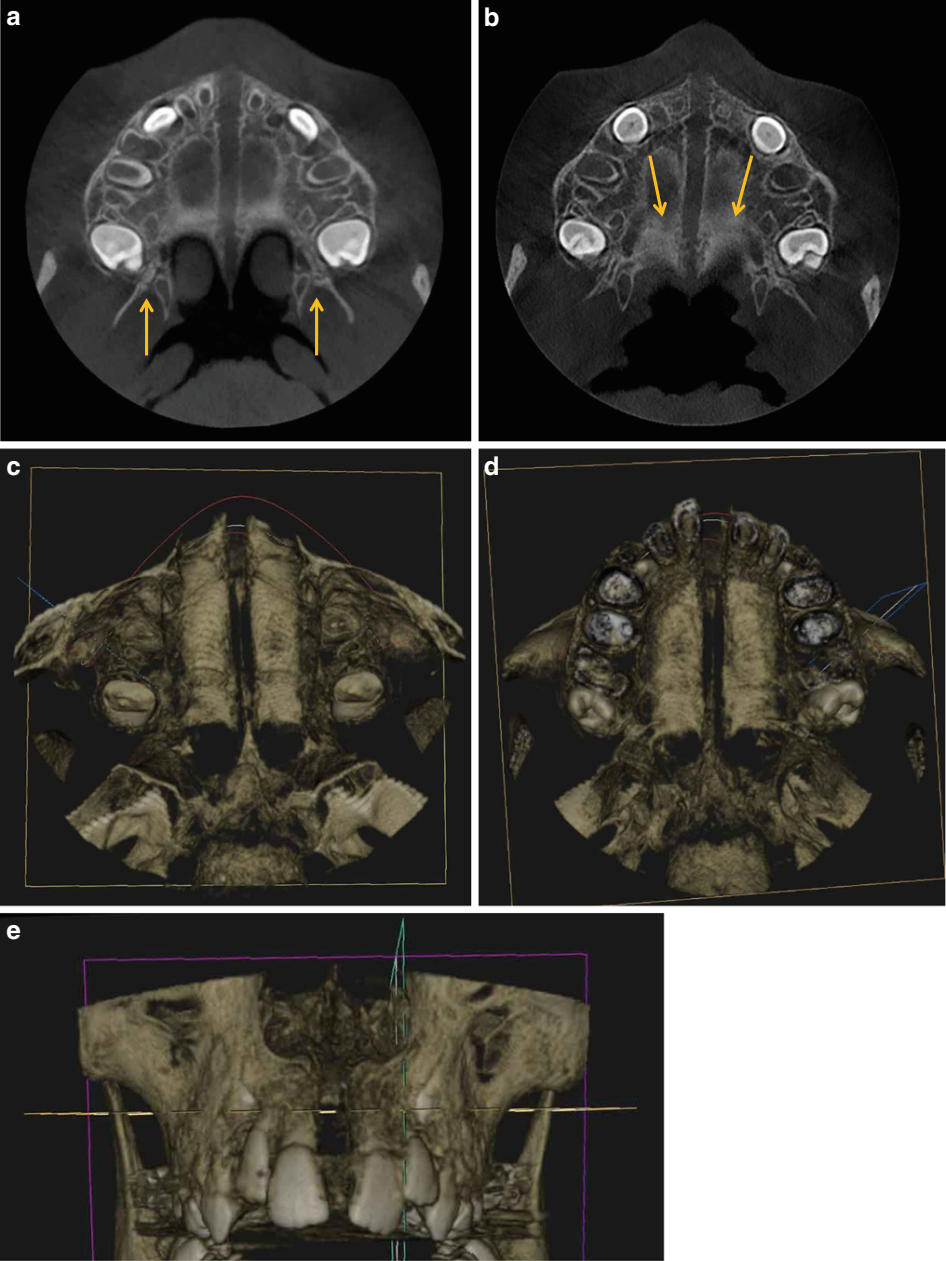
Horizontal slices (**a**,**b**) and horizontal and frontal three-dimensional reconstructions (**c**–**e**) after rapid maxillary expansion (RME) from the cone-beam computed tomography image of a 7.3-year-old girl (PG1). **a**,**b** Slices with open median and transverse (*yellow arrows*) sutures, on **a** additionally visible open pterygopalatomaxillary sutures (*yellow arrows*). **c**,**d** Parallel opening of the median palatine suture and open transverse palatine suture from cranial (**c**) and caudal (**d**). **e** Frontal view with parallel fully opened intermaxillary suture Horizontale Schichten (**a**,**b**) sowie horizontale und frontale 3‑D-Rekonstruktionen (**c**–**e**) nach RME („rapid maxillary expansion“) aus der DVT (digitale Volumentomographie) eines 7,3 Jahre alten Mädchens (PG 1). **a**,**b** Schichten mit offener medianer und transversaler (*gelbe Pfeile*) Gaumennaht, auf **a** zusätzlich sichtbare offene pterygopalatomaxilläre Suturen (*gelbe Pfeile*). **c**,**d** Parallele Öffnung der Sutura palatina mediana und offene Sutura palatina transversa von kranial (**c**) und kaudal (**d**). **e** Frontalansicht mit paralleler, vollständig geöffneter Sutura intermaxillaris

**Fig. 6:**
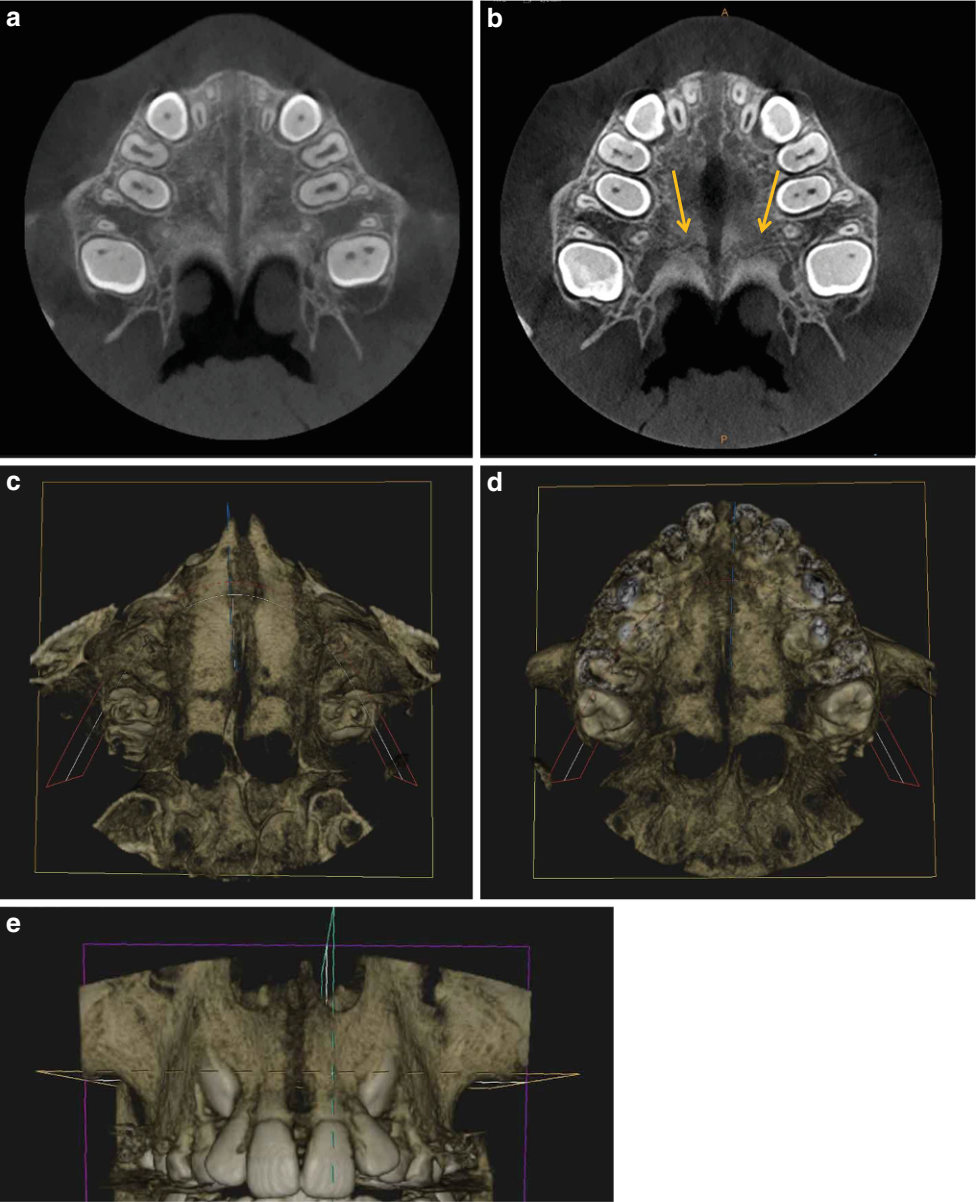
Horizontal slices (**a**,**b**) and horizontal and frontal three-dimensional reconstructions (**c**–**e**) after rapid maxillary expansion (RME) from the cone-beam computed tomography image of a 10.2-year-old boy (PG2). **a**,**b** Slices with open median and transverse (*yellow arrows*) sutures. The pterygopalatomaxillary sutures are closed. **c**,**d** Parallel opening of the median palatine suture and open transverse palatine suture from cranial (**c**) and caudal (**d**). **e** Frontal view with parallel opened intermaxillary suture. The openings are smaller despite identical screw activation Horizontale Schichten (**a**,**b**) sowie horizontale und frontale 3‑D-Rekonstruktionen (**c**–**e**) nach RME („rapid maxillary expansion“) aus der DVT (digitale Volumentomographie) eines 10,2 Jahre alten Jungen (PG 2). **a**,**b** Schichten mit offener medianer und transversaler (*gelbe Pfeile*) Gaumennaht. Die pterygopalatomaxillären Suturen sind geschlossen. **c**,**d** Parallele Öffnung der Sutura palatina mediana und offene Sutura palatina transversa von kranial (**c**) und kaudal (**d**). **e** Frontalansicht mit parallel geöffneter Sutura intermaxillaris. Die Öffnungen sind trotz identischer Schraubenaktivierung kleiner

**Fig. 7:**
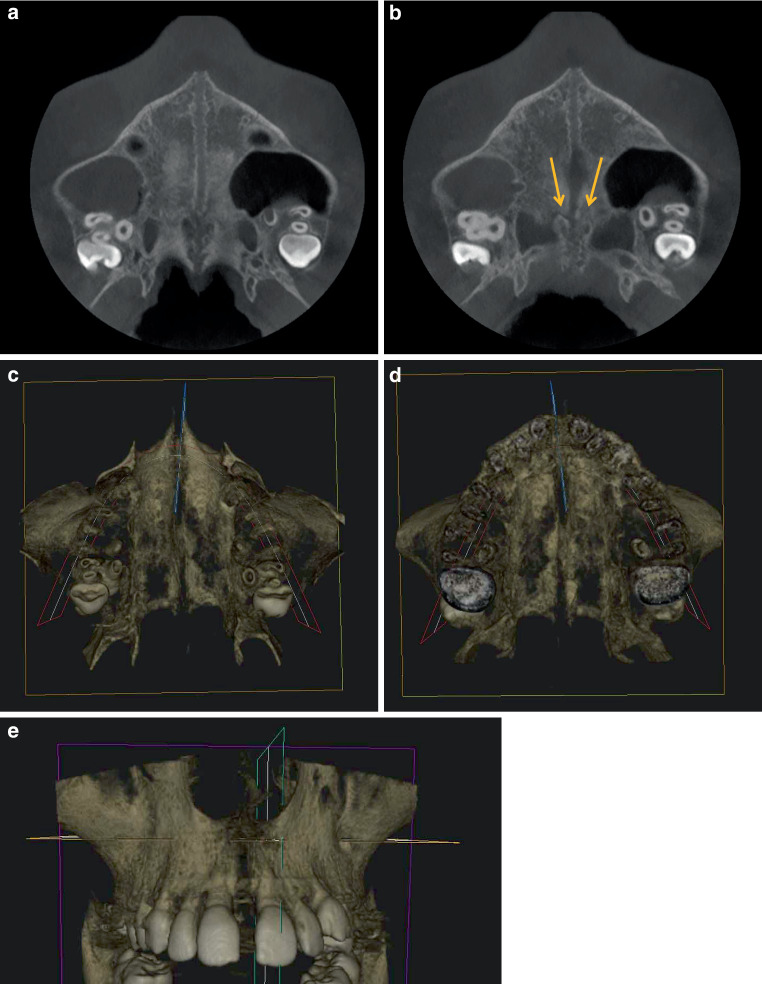
Horizontal slices (**a**,**b**) and horizontal and frontal three-dimensional reconstructions (**c**–**e**) after rapid maxillary expansion (RME) from the cone-beam computed tomography image of a 13.3-year-old boy (PG3). **a**,**b** slices with V‑shaped open median palatal and transverse sutures open only at their intersection (*yellow arrows*). The pterygopalatomaxillary sutures are closed. **c**,**d** V-shaped opening of the median palatine suture from cranial (**c**) and caudal (**d**). Transverse palatal suture visible to a limited extent. **e** Frontal view with V‑shaped opening of the intermaxillary suture from cranial to caudal Horizontale Schichten (**a**,**b**) und horizontale und frontale 3‑D-Rekonstruktionen (**c**–**e**) nach RME („rapid maxillary expansion“) aus der DVT (digitale Volumentomographie) eines 13,3-jährigen Jungen (PG 3). **a**,**b** Schnitte mit V‑förmig offener medianer und transversaler Gaumennaht, die nur an deren Schnittpunkt offen ist (*gelbe Pfeile*). Die pterygopalatomaxillären Suturen sind geschlossen. **c**,**d** V-förmige Öffnung der Sutura palatina mediana von kranial (**c**) und kaudal (**d**). Die Sutura palatina transversa ist in begrenztem Umfang sichtbar. **e** Frontalansicht mit von kranial nach kaudal V‑förmiger Öffnung der Sutura intermaxillaris

The CBCT scans of the patients from PG1 and PG2 show an almost parallel opening of the median palatal suture in the transverse plane wherever measured. However, the size of the opening is smaller in the patient from PG2 after an identical number of hyrax screw activations. It is striking that the paired pterygopalatomaxillary sutures were still partially open in the 7.3-year-old patient from PG1, but completely closed in the 10.2-year-old patient from PG2. The scan of the 13.3-year-old patient from PG3, on the other hand, shows a V-shaped opening of the median palatal suture which decreases from anterior to posterior, and closed pterygopalatomaxillary sutures. The age-dependant different expansion modes are also confirmed in the frontal views: parallel expansion of the intermaxillary sutures starting from the palate to the floor of the nose in the two younger patients from PG1 and PG2, but a triangular expansion with a cranially tapering opening in the older patient from PG3 (Figs. 5, 6 and 7).

The comparison of the three patients reveals fundamental differences in the behaviour of the transverse palatal suture after RME. In the two younger patients, a continuous opening of the transverse palatal suture is visible in the transverse plane on both sides. This is confirmed by the 3D reconstruction. In addition to the clearly opened intermaxillary and transverse palatal sutures, a bilaterally opened transverse palatal suture is visible in the transverse plane from both coronal and caudal sides. In the two younger patients from PG1 and PG2, the palatal sutures are consequently open in a cruciform shape and divide the hard palate into four areas. In the oldest patient from PG3, on the other hand, the transverse palatal suture cannot be depicted continuously in the scans of the horizontal plane. It only appears open over a short distance at the intersection with the median palatal suture. The 3D image shows a comparatively smaller, but triangular opening of the median palatal suture. Since the transverse palatal suture can only be visualised medially and appears obliterated laterally, the bony palate appears to be divided into two parts by the V‑shaped opened median palatal suture.

## Dental cast analysis

### Palatal width (transverse plane)

The palatal width increase is significant in all patients. In PG1, the increase is greater posteriorly than anteriorly at all levels. In contrast, a greater width increase occurred anteriorly than posteriorly in PG2 and PG3 at all levels. The amount of width increase within all groups decreases between the levels ascending from dental to skeletal–basal in almost all comparisons (Tables 2 and 3).

**Table 2:** Dental, gingival–alveolar, skeletal–basal widths (transverse plane), intragroup comparison Breite dental, gingivoalveolär, skelettal-basal (Transversalebene), Intragruppenvergleich

Measurement (mm)	T1 (M ± SD) 95% CI (LB, UB)	T2 (M ± SD) 95% CI (LB, UB)	∆T2 − T1 (M ± SD) 95% CI (LB, UB)	*p *(intra)
*All patients*				
54–64/14–24, dental	32.91 ± 1.79 32.45, 33.37	37.10 ± 2.22 36.53, 37.67	4.19 ± 1.85 3.71, 4.67	< 0.001 ***
54–64/14–24, GM, gingival	24.29 ± 1.40 23.92, 24.65	27.98 ± 1.50 27.60, 28.37	3.70 ± 1.58 3.29, 4.11	< 0.001 ***
54–64/14–24, GM, +2 mm	19.86 ± 1.62 19.44, 20.28	22.67 ± 1.91 22.17, 23.16	2.80 ± 1.51 2.41, 3.20	< 0.001 ***
54–64/14–24, GM, +4 mm	15.64 ± 2.14 15.09, 16.19	17.97 ± 2.19 17.41, 18.54	2.33 ± 1.50 1.95, 2.72	< 0.001 ***
54–64/14–24, GM, +6 mm	12.79 ± 2.55 12.13, 13.45	14.76 ± 2.41 14.13, 15.38	1.96 ± 1.54 1.56, 2.36	< 0.001 ***
16–26, dental	42.53 ± 2.26 41.95, 43.12	46.87 ± 2.48 46.23, 47.51	4.34 ± 1.58 3.93, 4.75	< 0.001 ***
16–26, GM gingival	31.50 ± 2.35 30.89, 32.11	35.03 ± 2.90 34.28, 35.78	3.53 ± 1.74 3.08, 3.98	< 0.001 ***
16–26, GM +2 mm	26.72 ± 2.24 26.14, 27.30	28.88 ± 2.34 28.28, 29.49	2.16 ± 1.71 1.72, 2.60	< 0.001 ***
16–26, GM +4 mm	24.00 ± 2.42 23.38, 24.63	25.76 ± 2.68 25.07, 26.46	1.76 ± 1.92 1.27, 2.26	< 0.001 ***
16–26, GM +6 mm	20.46 ± 3.17 19.65, 21.28	22.16 ± 3.45 21.27, 23.05	1.70 ± 2.12 1.15, 2.25	< 0.001 ***
*Group 1*				
54–64/14–24, dental	32.25 ± 2.02 31.30, 33.19	35.66 ± 1.87 34.79, 36.54	3.41 ± 1.88 2.54, 4.29	< 0.001 ***
54–64/14–24, GM, gingival	24.31 ± 1.69 23.52, 25.11	27.49 ± 1.56 26.76, 28.22	3.18 ± 1.71 2.38, 3.98	< 0.001 ***
54–64/14–24, GM, +2 mm	19.38 ± 2.03 18.43, 20.33	21.78 ± 2.11 20.79, 22.77	2.40 ± 1.46 1.72, 3.08	< 0.001 ***
54–64/14–24, GM, +4 mm	14.87 ± 2.24 13.82, 15.92	17.04 ± 2.11 16.05, 18.03	2.17 ± 1.22 1.60, 2.74	< 0.001 ***
54–64/14–24, GM, +6 mm	12.31 ± 2.57 11.10, 13.51	14.02 ± 2.16 13.01, 15.04	1.72 ± 1.37 1.08, 2.36	< 0.001 ***
16–26, dental	42.46 ± 2.34 41.36, 43.55	47.15 ± 2.43 46.01, 48.28	4.69 ± 1.72 3.89, 5.50	< 0.001 ***
16–26, GM gingival	32.01 ± 2.57 30.81, 33.22	36.15 ± 2.92 34.78, 37.52	4.14 ± 1.83 3.28, 4.99	< 0.001 ***
16–26, GM +2 mm	26.56 ± 2.60 25.34, 27.78	29.34 ± 2.82 28.02, 30.66	2.78 ± 2.16 1.77, 3.79	< 0.001 ***
16–26, GM +4 mm	23.02 ± 2.93 21.65, 24.39	25.35 ± 3.50 23.71, 26.99	2.33 ± 2.43 1.19, 3.47	< 0.001 ***
16–26, GM +6 mm	18.30 ± 3.46 16.68, 19.92	20.63 ± 4.19 18.66, 22.59	2.33 ± 2.55 1.13, 3.52	< 0.001 ***
*Group 2*				
54–64/14–24, dental	32.83 ± 1.35 32.20, 33.46	37.68 ± 1.64 36.91, 38.45	4.85 ± 1.58 4.11, 5.58	< 0.001 ***
54–64/14–24, GM, gingival	24.34 ± 1.15 23.80, 24.88	28.26 ± 1.10 27.75, 28.78	3.93 ± 1.14 3.39, 4.46	< 0.001 ***
54–64/14–24, GM, +2 mm	19.98 ± 1.62 19.22, 20.74	22.94 ± 1.40 22.28, 23.60	2.96 ± 1.39 2.31, 3.61	< 0.001 ***
54–64/14–24, GM, +4 mm	16.09 ± 2.18 15.07, 17.11	18.38 ± 1.88 17.50, 19.26	2.29 ± 1.63 1.53, 3.06	< 0.001 ***
54–64/14–24, GM, +6 mm	13.18 ± 2.50 12.01, 14.36	15.06 ± 2.28 14.00, 16.13	1.88 ± 1.61 1.13, 2.63	< 0.001 ***
16–26, dental	42.78 ± 1.64 42.01, 43.55	47.46 ± 1.61 46.71, 48.22	4.68 ± 1.03 4.19, 5.16	< 0.001 ***
16–26, GM gingival	31.62 ± 1.51 30.92, 32.33	35.36 ± 1.72 34.55, 36.16	3.73 ± 1.48 3.04, 4.43	< 0.001 ***
16–26, GM +2 mm	27.05 ± 1.68 26.26, 27.83	28.85 ± 1.56 28.12, 29.58	1.81 ± 1.34 1.18, 2.43	< 0.001 ***
16–26, GM +4 mm	24.36 ± 1.70 23.56, 25.15	25.66 ± 1.98 24.74, 26.59	1.31 ± 1.87 0.43, 2.18	0.006 **
16–26, GM +6 mm	20.99 ± 2.10 20.01, 21.97	22.08 ± 2.69 20.82, 23.33	1.09 ± 2.28 0.02, 2.15	0.046 *
*Group 3*				
54–64/14–24, dental	33.65 ± 1.75 32.83, 34.47	37.96 ± 2.41 36.83, 39.09	4.31 ± 1.88 3.43, 5.19	< 0.001 ***
54–64/14–24, GM, gingival	24.21 ± 1.39 23.56, 24.86	28.20 ± 1.72 27.39, 29.00	3.99 ± 1.77 3.16, 4.82	< 0.001 ***
54–64/14–24, GM, +2 mm	20.23 ± 1.01 19.76, 20.70	23.28 ± 1.91 22.39, 24.17	3.05 ± 1.67 2.27, 3.83	< 0.001 ***
54–64/14–24, GM, +4 mm	15.96 ± 1.87 15.08, 16.83	18.50 ± 2.36 17.39, 19.61	2.54 ± 1.67 1.76, 3.33	< 0.001 ***
54–64/14–24, GM, +6 mm	12.89 ± 2.63 11.66, 14.12	15.18 ± 2.71 13.91, 16.45	2.29 ± 1.66 1.51, 3.07	< 0.001 ***
16–26, dental	42.36 ± 2.75 41.07, 43.64	46.01 ± 3.06 44.58, 47.44	3.65 ± 1.74 2.84, 4.46	< 0.001 ***
16–26, GM gingival	30.85 ± 2.76 29.56, 32.14	33.57 ± 3.31 32.02, 35.12	2.72 ± 1.66 1.94, 3.49	< 0.001 ***
16–26, GM +2 mm	26.56 ± 2.40 25.44, 27.68	28.46 ± 2.51 27.29, 29.64	1.91 ± 1.41 1.25, 2.56	< 0.001 ***
16–26, GM +4 mm	24.63 ± 2.28 23.57, 25.70	26.28 ± 2.38 25.17, 27.39	1.65 ± 1.20 1.09, 2.21	< 0.001 ***
16–26, GM +6 mm	22.10 ± 2.60 20.89, 23.31	23.78 ± 2.62 22.55, 25.00	1.68 ± 1.24 1.10, 2.26	< 0.001 ***

Widths (in mm) in the anterior (54–64/14–24) and posterior (16–26) regions at five different levels of the maxilla. The dental width, the gingival/alveolar width, the width 2, 4 and 6 mm cranial to the gingival/alveolar level are shown

*M* Mean, *SD* standard deviation, *CI* confidence intervals and significance levels, *NS* not significant, *GM* gingival margin, patient groups PG 1, PG 2 and PG 3

**Table 3:** Dental, gingival–alveolar, skeletal–basal widths (transverse plane), intergroup comparison Breite dental, gingivoalveolär, skelettal-basal (Transversalebene), Intergruppenvergleich

Measurement (mm)	Intergroup comparison (*p* inter)		
	PG 1 vs PG 2	PG1 vs PG 3	PG 2 vs 3
54–64/14–24, dental	0.037 *	0.258 ^NS^	0.614 ^NS^
54–64/14–24, GM, gingival	0.293 ^NS^	0.238 ^NS^	0.991 ^NS^
54–64/14–24, GM, +2 mm	0.474 ^NS^	0.367 ^NS^	0.980 ^NS^
54–64/14–24, GM, +4 mm	0.965 ^NS^	0.720 ^NS^	0.861 ^NS^
54–64/14–24, GM, +6 mm	0.942 ^NS^	0.478 ^NS^	0.682 ^NS^
16–26, dental	1.000 ^NS^	0.089 ^NS^	0.094 ^NS^
16–26, GM gingival	0.725 ^NS^	0.024 *	0.138 ^NS^
16–26, GM +2 mm	0.169 ^NS^	0.235 ^NS^	0.981 ^NS^
16–26, GM +4 mm	0.214 ^NS^	0.498 ^NS^	0.839 ^NS^
16–26, GM +6 mm	0.158 ^NS^	0.595 ^NS^	0.647 ^NS^

Significance levels of intergroup comparisons in the transverse plane, *NS* not significant, *GM* gingival margin, patient groups PG 1, PG 2 and PG 3

## Palatal depth: ratio of anterior to posterior width (sagittal plane)

The width ratio a/p, i.e. the quotient of the width differences between T1 and T2, was determined on the three defined levels (dental, gingival–alveolar, skeletal–basal). In PG1, there is a parallel to slightly dorsal V‑shaped transverse expansion on all levels. The ratio of the palatal width from anterior to posterior thus remained largely unchanged in PG1. The situation is different in PG2 and especially in PG3. Here, the increase in palatal width is significantly greater anteriorly than posteriorly at all levels (PG2: *p* < 0.001, *p* = 0.006, *p* = 0.009; PG3: *p* = 0.003, *p* < 0.001, *p* = 0.002) and thus occurs in a ventral V‑shape. This is particularly pronounced in PG3 at the gingival–alveolar and skeletal–basal levels. Accordingly, the differences between PG1 and PG3 are significant at all levels (*p* < 0.001, *p* = 0.022, *p* = 0.021; Tables 4 and 5).

**Table 4:** Ratio of anterior to posterior width (sagittal plane) at different timepoints, intragroup comparison Ratio der Breite anterior zu posterior (Gaumentiefe, Sagittalebene), Intragruppenvergleich

Measurement (mm)	T1 (M ± SD) 95% CI (LB, UB)	T2 (M ± SD) 95% CI (LB, UB)	∆T2 − T1 (M ± SD) 95% CI (LB, UB)	∆T2 − T1 (Diff.) (M ± SD) 95% CI (LB, UB)	*p* (intra)
*All patients*					
Ratio a/p dental	0.77 ± 0.04 0.76, 0.79	0.79 ± 0.05 0.78, 0.81	0.02 ± 0.04 0.01, 0.03	1.06 ± 0.60 0.90, 1.22	< 0.001 ***
Ratio a/p GM, Gingival–alveolar	0.77 ± 0.06 0.76, 0.79	0.80 ± 0.07 0.79, 0.82	0.03 ± 0.05 0.02, 0.04	1.41 ± 1.45 1.04, 1.79	< 0.001 ***
Ratio a/p + 6 mm, Skeletal–basal	0.64 ± 0.13 0.60, 0.67	0.68 ± 0.12 0.64, 0.71	0.04 ± 0.09 0.02, 0.06	1.64 ± 2.92 0.88, 2.39	0.001 **
*Group 1*					
Ratio a/p dental	0.76 ± 0.04 0.74, 0.78	0.76 ± 0.04 0.74, 0.78	0.00 ± 0.03 −0.02, 0.01	0.73 ± 0.33 0.57, 0.89	0.749 ^NS^
Ratio a/p GM, Gingival–alveolar	0.76 ± 0.05 0.74, 0.78	0.76 ± 0.05 0.74, 0.79	0.00 ± 0.04 −0.02, 0.02	0.81 ± 0.41 0.61, 1.00	0.778 ^NS^
Ratio a/p + 6 mm, Skeletal–basal	0.69 ± 0.14 0.62, 0.75	0.70 ± 0.13 0.63, 0.76	0.01 ± 0.11 −0.04, 0.06	0.70 ± 0.58 0.43, 0.97	0.636 ^NS^
*Group 2*					
Ratio a/p dental	0.77 ± 0.03 0.75, 0.78	0.79 ± 0.04 0.78, 0.81	0.03 ± 0.03 0.01, 0.04	1.04 ± 0.30 0.90, 1.18	< 0.001 ***
Ratio a/p GM, Gingival–alveolar	0.77 ± 0.05 0.75, 0.79	0.80 ± 0.05 0.78, 0.83	0.03 ± 0.04 0.01, 0.05	1.43 ± 1.55 0.70, 2.15	0.006 **
Ratio a/p + 6 mm, Skeletal–basal	0.63 ± 0.12 0.58, 0.69	0.69 ± 0.11 0.64, 0.74	0.06 ± 0.09 0.02, 0.10	1.10 ± 0.67 0.78, 1.41	0.009 **
*Group 3*					
Ratio a/p dental	0.80 ± 0.05 0.78, 0.82	0.83 ± 0.05 0.80, 0.85	0.03 ± 0.04 0.01, 0.05	1.41 ± 0.83 0.97, 1.46	0.003 **
Ratio a/p GM, Gingival–alveolar	0.79 ± 0.07 0.73, 0.84	0.85 ± 0.07 0.81, 0.88	0.05 ± 0.06 0.04, 0.10	2.01 ± 1.79 0.94, 1.89	< 0.001 ***
Ratio a/p + 6 mm, Skeletal–basal	0.59 ± 0.14 0.53, 0.68	0.64 ± 0.12 0.64, 0.72	0.05 ± 0.07 0.03, 0.12	3.11 ± 4.70 0.80, 3.00	0.002 **

Ratio a/p of the width on the dental, gingival-alveolar and skeletal-basal plane. ∆T2 − T1 (Diff.) was determined from the ratio of the differences of the respective widths in the anterior and posterior area between T1 and T2. $\frac{\text{Width}\:\mathrm{a}\mathrm{T}2-\text{Width}\:\mathrm{a}\mathrm{T}1}{\text{Width}\:\mathrm{p}\mathrm{T}2-\text{Width}\:\mathrm{p}\mathrm{T}1}$ ∆T2 − T1 (Diff.) < 1 indicates a greater increase in the posterior region, ∆T2 − T1 (Diff.) = 1 shows an equal change anteriorly and posteriorly, ∆T2 − T1 (Diff.) > 1 indicates a greater increase in the anterior region

*M* Mean, *SD* standard deviation, *CI* confidence intervals and significance levels, *NS* not significant, *a* anterior (first deciduous or premolar), *p* posterior (first molar), *GM* gingival margin

**Table 5:** Ratio of anterior to posterior width (sagittal plane), intergroup comparison Ratio der Breite anterior zu posterior (Gaumentiefe, Sagittalebene), Intergruppenvergleich

Measurement (mm)	Intergroup comparison (*p* inter)		
	PG 1 vs PG 2	PG1 vs PG 3	PG 2 vs 3
Ratio a/p dental	0.175 ^NS^	< 0.001 ***	0.095 ^NS^
Ratio a/p GM, Gingival–alveolar	0.340 ^NS^	0.022 *	0.386 ^NS^
Ratio a/p + 6 mm, Skeletal–basal	0.894 ^NS^	0.021 *	0.063 ^NS^

Significance levels of intergroup comparisons in the sagittal plane, *NS* not significant, *a* anterior (first deciduous or premolar), *p* posterior (first molar), *GM* gingival margin

## Palatal height (frontal plane)

In PG 1, the height at each measurement point increases significantly both anteriorly and posteriorly. In the two older patients (PG 2 and PG 3), however, with one exception (PG 3: 5 mm left lateral), the change in posterior height is insignificant. In the sagittal, anterioposterior comparison, the height increase in the older patients (PG 2 and PG 3) occurs more pronounced anteriorly than posteriorly. In PG 1, on the other hand, the median height increase is almost the same. It is striking that almost everywhere the height increases are greater on the right and left sides than directly at the raphe median line. There are no significant differences in the palatal height change between the groups (Tables 6 and 7).

**Table 6:** Height (frontal plane) at different timepoints, intragroup comparison Höhe (Frontalebene) zu verschiedenen Zeitpunkten, Intragruppenvergleich

Measurement (mm)	T1 (M ± SD) 95% CI (LB, UB)	T2 (M ± SD) 95% CI (LB, UB)	∆T2 − T1 (M ± SD) 95% CI (LB, UB)	*p *(intra)
*All patients*				
RML ant median	10.24 ± 1.85 9.77, 10.72	11.02 ± 2.00 10.50, 11.53	0.77 ± 1.19 0.47, 1.08	< 0.001 ***
RML ant 5 mm right	8.01 ± 2.08 7.47, 8.55	9.17 ± 2.33 8.57, 9.78	1.16 ± 1.39 0.81, 1.52	< 0.001 ***
RML ant 5 mm left	7.96 ± 2.21 7.39, 8.53	8.92 ± 2.27 8.34, 9.51	0.97 ± 1.47 0.59, 1.34	< 0.001 ***
RML post median	13.38 ± 2.46 12.74, 14.01	13.77 ± 2.69 13.08, 14.46	0.39 ± 0.99 0.14, 0.65	0.003 **
RML post 5 mm right	11.83 ± 2.47 11.19, 12.47	12.30 ± 2.53 11.65, 12.95	0.47 ± 1.15 0.17, 0.77	0.002 **
RML post 5 mm left	11.95 ± 2.46 11.31, 12.58	12.53 ± 2.78 11.81, 13.25	0.58 ± 1.28 0.25, 0.91	< 0.001 ***
*Group 1*				
RML ant median	9.90 ± 1.60 9.15, 10.65	10.49 ± 1.75 9.67, 11.31	0.59 ± 1.07 0.09, 1.09	0.023 *
RML ant 5 mm right	7.44 ± 1.86 6.57, 8.31	8.45 ± 2.14 7.44, 9.45	1.01 ± 1.50 0.31, 1.71	0.007 **
RML ant 5 mm left	7.29 ± 1.73 6.48, 8.10	8.47 ± 1.79 7.63, 9.30	1.18 ± 1.35 0.55, 1.81	< 0.001 ***
RML post median	11.72 ± 1.52 11.00, 12.43	12.25 ± 1.82 11.40, 13.10	0.53 ± 1.12 0.01, 1.05	0.047 *
RML post 5 mm right	10.16 ± 1.97 9.24, 11.08	10.85 ± 1.84 9.99, 11.71	0.69 ± 1.36 0.05, 1.33	0.036 *
RML post 5 mm left	10.18 ± 1.81 9.33, 11.03	10.82 ± 2.20 9.79, 11.85	0.64 ± 1.19 0.09, 1.20	0.026 *
*Group 2*				
RML ant median	10.30 ± 2.23 9.26, 11.34	11.15 ± 2.68 9.90, 12.40	0.85 ± 1.20 0.29, 1.41	0.005 **
RML ant 5 mm right	8.41 ± 2.36 7.31, 9.52	9.71 ± 2.87 8.36, 11.05	1.29 ± 1.43 0.62, 1.96	< 0.001 ***
RML ant 5 mm left	8.25 ± 2.58 7.05, 9.46	9.09 ± 2.88 7.74, 10.44	0.84 ± 1.51 0.13, 1.54	0.023 *
RML post median	13.67 ± 3.03 12.25, 15.08	13.96 ± 3.35 12.39, 15.53	0.30 ± 1.01 −0.18, 0.77	0.206 ^NS^
RML post 5 mm right	12.13 ± 2.72 10.86, 13.40	12.49 ± 3.06 11.05, 13.92	0.36 ± 1.25 −0.23, 0.94	0.217 ^NS^
RML post 5 mm left	12.14 ± 2.49 10.98, 13.30	12.55 ± 3.10 11.10, 14.00	0.41 ± 1.49 −0.28, 1.11	0.230 ^NS^
*Group 3*				
RML ant median	10.53 ± 1.69 9.55, 11.27	11.41 ± 1.30 10.62, 12.47	0.88 ± 1.33 0.25, 1.38	0.008 **
RML ant 5 mm right	8.17 ± 1.96 7.26, 9.00	9.37 ± 1.77 8.54, 10.53	1.19 ± 1.28 0.66, 1.79	< 0.001 ***
RML ant 5 mm left	8.34 ± 2.20 7.23, 9.53	9.22 ± 2.05 8.46, 10.49	0.88 ± 1.59 0.22, 1.84	0.023 *
RML post median	14.75 ± 1.53 14.13, 15.35	15.10 ± 1.87 13.35, 15.76	0.35 ± 0.88 −0.08, 0.80	0.089 ^NS^
RML post 5 mm right	13.20 ± 1.61 12.39, 14.23	13.57 ± 1.78 12.41, 15.16	0.37 ± 0.79 0.13, 0.78	0.051 ^NS^
RML post 5 mm left	13.52 ± 1.84 12.25, 14.36	14.21 ± 1.88 12.82, 15.10	0.69 ± 1.18 −0.13, 1.85	0.017 *

Heights (in mm) of the palate. In the anterior region, median (raphe median line) and 5 mm right and left paramedian were measured; in the posterior region, median (raphe median line) and 5 mm and 10 mm right and left paramedian were measured

*M* Mean, *SD* standard deviation, *CI* confidence intervals and significance levels, *NS* not significant, *RML* raphe median line, *ri* right (first quadrant), *le* left (second quadrant), *ant* anterior (first deciduous or premolar), *post* posterior (first molar)

**Table 7:** Height (frontal plane), intergroup comparison Höhe (Frontalebene), Intergruppenvergleich

Measurement (mm)	Intergroup comparison (*p* inter)		
	PG 1 vs PG 2	PG1 vs PG 3	PG 2 vs 3
RML ant median	0,781 ^NS^	0,735 ^NS^	0,997 ^NS^
RML ant 5 mm right	0,795 ^NS^	0,908 ^NS^	0,972 ^NS^
RML ant 5 mm left	0,750 ^NS^	0,800 ^NS^	0,996 ^NS^
RML post median	0,743 ^NS^	0,840 ^NS^	0,984 ^NS^
RML post 5 mm right	0,638 ^NS^	0,655 ^NS^	1,000 ^NS^
RML post 5 mm left	0,838 ^NS^	0,993 ^NS^	0,775 ^NS^

Significance levels of intergroup comparisons in the frontal plane (height), *NS* not significant, *RML* raphe median line, *ri* right (first quadrant), *le* left (second quadrant), *ant* anterior (first deciduous or premolar), *post* posterior (first molar)

## Palatal depth: ratio of anterior to posterior height (sagittal plane)

The height ratio a/p, i.e. the quotient of the height differences between T1 and T2, was determined on three sagittal planes. In all patients and in all groups, the height changes were more pronounced anteriorly than posteriorly. An increase of the ratio occurred from PG 1 to PG 2 to PG 3, both median and 5 mm right and left paramedian. The results of the height measurements allow the conclusion that reduced height changes with increasing age in the posterior region are the reason for this. Since the intergroup comparison revealed significant changes in only two places, the results are to be interpreted as tendencies (Tables 8 and 9).

**Table 8:** Ratio of anterior to posterior height (sagittal plane), intragroup comparison Ratio der Höhe anterior zu posterior (Sagittalebene), Intragruppenvergleich

Measurement (mm)	T1 (M ± SD) 95% CI (LB, UB)	T2 (M ± SD) 95% CI (LB, UB)	∆T2 − T1 (M ± SD) 95% CI (LB, UB)	∆T2 − T1 (Diff.) (M ± SD) 95% CI (LB, UB)	*p* (intra)
*All patients*					
Ratio a/p RML	0.78 ± 0.11 0.75, 0.80	0.81 ± 0.13 0.78, 0.85	0.04 ± 0.09 0.01, 0.06	1.99 ± 1.42 1.62, 2.35	0.003 **
Ratio a/p RML 5 mm ri	0.69 ± 0.17 0.65, 0.74	0.76 ± 0.17 0.71, 0.80	0.07 ± 0.13 0.03, 0.10	2.06 ± 1.83 1.58, 2.53	< 0.001 ***
Ratio a/p RML 5 mm le	0.67 ± 0.15 0.63, 0.71	0.73 ± 0.16 0.69, 0.77	0.05 ± 0.12 0.02, 0.08	1.87 ± 1.52 1.48, 2.27	0.001 **
*Group 1*					
Ratio a/p RML	0.85 ± 0.09 0.80, 0.89	0.86 ± 0.11 0.81, 0.91	0.01 ± 0.10 −0.04, 0.06	1.58 ± 1.30 0.96, 2.19	0.564 ^NS^
Ratio a/p RML 5 mm ri	0.74 ± 0.17 0.66, 0.82	0.78 ± 0.17 0.70, 0.86	0.04 ± 0.16 −0.04, 0.11	1.30 ± 1.47 0.61, 1.99	0.324 ^NS^
Ratio a/p RML 5 mm le	0.72 ± 0.13 0.66, 0.78	0.79 ± 0.11 0.74, 0.84	0.07 ± 0.14 0.01, 0.14	1.14 ± 0.81 0.76, 1.52	0.031 *
*Group 2*					
Ratio a/p RML	0.76 ± 0.13 0.69, 0.88	0.82 ± 0.15 0.69, 0.92	0.05 ± 0.10 0.02, 0.10	1.98 ± 1.30 1.13, 2.94	0.030 *
Ratio a/p RML 5 mm ri	0.71 ± 0.18 0.57, 0.80	0.80 ± 0.19 0.71, 0.94	0.09 ± 0.12 0.10, 0.15	2.26 ± 2.19 0.88, 2.81	0.005 **
Ratio a/p RML 5 mm le	0.69 ± 0.18 0.56, 0.80	0.74 ± 0.18 0.62, 0.87	0.05 ± 0.11 0.00, 0.09	1.59 ± 1.28 0.57, 2.30	0.074 ^NS^
*Group 3*					
Ratio a/p RML	0.71 ± 0.08 0.67, 0.75	0.76 ± 0.10 0.72, 0.81	0.05 ± 0.08 0.01, 0.08	2.41 ± 1.59 1.66, 3.15	0,013 *
Ratio a/p RML 5 mm ri	0.63 ± 0.15 0.56, 0.69	0.70 ± 0.14 0.63, 0.76	0.07 ± 0.09 0.03, 0.12	2.61 ± 1.58 1.87, 3.35	0.002 **
Ratio a/p RML 5 mm le	0.62 ± 0.14 0.55, 0.68	0.65 ± 0.15 0.58, 0.72	0.04 ± 0.10 −0.01, 0.09	2.89 ± 1.78 2.06, 3.72	0.129 ^NS^

Ratio a/p of the anterior-posterior palatal height at the median palatal suture, and 5 mm left or right paramedian. ∆T2 − T1 (Diff.) was determined from the ratio of the differences of the respective widths in the anterior and posterior area between T1 and T2. $\frac{\text{Height}\:\mathrm{a}\mathrm{T}2-\text{Height}\:\mathrm{a}\mathrm{T}1}{\text{Height}\:\mathrm{p}\mathrm{T}2-\text{Height}\:\mathrm{p}\mathrm{T}1}$ ∆T2 − T1 (Diff.) < 1 indicates a greater increase in the posterior region, ∆T2 − T1 (Diff.) = 1 shows an equal change anteriorly and posteriorly, ∆T2 − T1 (Diff.) > 1 indicates a greater increase in the anterior region

*M* Mean, *SD* standard deviation, *CI* confidence intervals and significance levels, *NS* not significant, *RML* raphe median line, *ri* right (first quadrant), *le* left (second quadrant), *a* anterior (first deciduous or premolar), *p* posterior (first molar), *RML* gingival margin

**Table 9:** Ratio of anterior to posterior height (sagittal plane), intergroup comparison Ratio der Höhe anterior zu posterior (Sagittalebene), Intergruppenvergleich

Measurement (mm)	Intergroup comparison (*p* inter)		
	PG 1 vs PG 2	PG1 vs PG 3	PG 2 vs 3
Ratio a/p RML	0.637 ^NS^	0.155 ^NS^	0.601 ^NS^
Ratio a/p RML 5 mm ri	0.206 ^NS^	0.058 ^NS^	0.808 ^NS^
Ratio a/p RML 5 mm le	0.538 ^NS^	< 0.001 ***	0.010 *

Significance levels of intergroup comparisons in the sagittal plane, *NS* not significant, *RML* raphe median line, *ri* right (first quadrant), *le* left (second quadrant), *a* anterior (first deciduous or premolar), *p* posterior (first molar), *RML* gingival margin

## Discussion

### Congruence of CBTC scan and dental cast analyses results

With 9 patients, it is possible to correlate the results of a CBCT scan and a dental cast analysis. Their age distribution allowed to allocate these patients evenly to the three groups of the extensive cast study, allowing insights into age-progressive sutural and morphological changes of the palate.

Many CBCT studies with measurements of the median palatal suture use measurement distances other than those used here, e.g., anterior to posterior nasal spine, ANS/PNS [10, 12, 26, 31]. In the present study, the suture was deliberately measured at the locations shown in Fig. 1a. As previously reported [14, 33, 43], a projection of the transverse distances of the anchorage teeth onto the suture plane was used to also allow direct comparability with the results of the cast measurement.

In the transverse plane, there is congruence between the values found in the cast analysis and the median palatal suture measurement (Fig. 4b, Table 1). Despite individual variability, the results of a CBCT scan analysis with those of the cast analysis in PG1 patients allow the deduction that rapid maxillary expansion causes nearly parallel opening of the median palatal suture and parallel distancing of the tooth-bearing palate until the age of 10. Parallel suture opening in patients with a chronological age of 10 years or less is also described in studies by Christie et al. [8], Habersack et al. [16] and Podesser et al. [33]. With increasing age, however, RME causes the median palatal suture to open increasingly in a triangular manner, as also described by other authors [3, 10, 16, 43]. In the transverse plane, a relatively greater width increase occurs anteriorly than posteriorly in the older patients from PG2 and PG3. However, the reason for this is the declining increase towards the posterior region. For the sagittal plane this means a continuous, V‑shaped change of the palate.

In the frontal plane, the measurement of the suture shows the same tendency as in the transverse plane.

The detailed dental cast analysis also shows that the palate height increases more on the right and left sides paramedial than on the raphe median line, and that this occurs more anteriorly than posteriorly in all patient groups. Especially in the older patients from PG2 and PG3, the change in height posteriorly is very small and, with one exception, not significant.

## Age-related structural changes of the maxillary sutures

The forces and moments generated by hyrax screw activation act not only on the median palatal suture but also on the surrounding sutures [4, 14, 21, 22, 35, 37], especially the palatine bones and the pterygoid process of the sphenoid bone. The tensions are initially concentrated on the anterior palate, then run dorsally along the median palatal suture and via the palatine bone to the sphenoid bone, the zygomatic process and the medial orbital walls [7]. The forces generated after therapeutic opening of the median palatal suture do not drop significantly, allowing the conclusion that the main resistance to palatal expansion lies not in the median palatal suture itself but in the surrounding maxillary connections [19, 46]. However, different, age-related structural changes must be considered.

In the case of the median palatal suture, it has been possible to qualify and quantify postnatal development based on histological studies of human specimens [24, 28, 32, 42]. Melsen [28] divided the sutural ossification process from birth to the age of 18 years into three phases. With increasing age, she describes a reduction in sutural width with increasing meandering interlocking of the two palatal processes. Further histological examinations of human palates prove an age-dependent obliteration process from posterior to anterior with generally very low obliteration values, but with strong inter- and intraindividual variations [24, 25, 32, 42].

The transverse palatine suture separates the palatine process of the maxillary bone from the horizontal plate of the palatine bone and crosses the median palatine suture in its dorsal region. In 1977, Persson and Thilander [32] found in a histological study of human specimens that the transverse palatine suture begins to obliterate later than the posterior, but earlier than the anterior part of the median palatal suture. However, the extent of obliteration seems to be less in a direct age comparison.

In 1982, Melsen and Melsen [29] also described the postnatal development of the palatomaxillary region from the newborn to the age of 27 in a human cadaver study. They found a change in morphology of the transverse palatal suture occurring before and during pubertal growth. A slightly wavy course develops into a distinct squamous (overlapping) suture.

Tschechne [40] was able to specify these age-related morphological changes of transverse palatal suture based on a study with 155 human skulls in 2005: He found that the position of the transverse palatal suture changes with age in both sagittal and vertical directions. While the maxilla moves ventrally, the transverse palatal suture develops a bone deficit. Its sutural growth then fills the bone deficit caused by maxillary displacement. This sutural growth initially takes place on the ventral, maxillary side. Until the age of 7, this growth is five times as large as on the palatal side, until the age of 12 it is still 2.5 times as large. In the next few years, sutural growth changes both qualitatively and quantitatively: by the age of 19, the maxillary side of the suture grows only one-tenth as much as the palatal side. In addition, sutural growth between 12 and 19 years represents only about 20% of the total growth until the 19th year of age. It can be assumed that the markedly reduced growth rate from the age of 12 increases the tendency to obliterate and thus the rigidity of the transverse palatal suture significantly. This is also evident in the oldest patient with CBCT scan in this study.

Timms [39] described the anatomical proximity of the paired palatine processes of the maxilla and palatine bone with the pterygoid process of the sphenoid bone. Consequently, he investigated a possible age-dependent correlation between expansion of the dentoalveolar arch and the pterygoid hamulus by intraoral measurements. Timms found that the pterygoid processes of the sphenoid bone spread laterally outwards in the lower region. Despite only a weak correlation, there was a tendency for increasing age to be a factor in the gradual reduction of this basic movement.

However, Ghoneima et al. [14] found no significant changes in pterygopalatomaxillary sutures after RME treatment in 20 patients aged between 8 and 15 years in a CT study. This is also evident in the CBCT scans evaluated in this study. In 8 patients between 8 and 14 years, the pterygopalatomaxillary sutures are bilaterally closed. Only the youngest patient, 7.3 years old, shows partially open sutures (Table 1, Fig. 5a).

Melsen and Melsen [29] described the role of the pterygopalatine sutures in preventing posterior expansion and concluded that this suture limits the extent of expansion and dictates the expansion pattern under RME treatment.

From the results of the studies by Timms [39], Ghoneima et al. [14] and Melsen and Melsen [29], the different findings of the median palatal sutures in relation to the obliteration findings of the pterygopalatomaxillary sutures can thus be explained, especially from the first compared to the second, but also to the third patient example of this study.

Thus, the morphology of the median palatal suture itself does not appear to have a limiting factor on the opening mode. The results of the present clinical study combined with the radiological case studies indicate that the age-related changes in the transverse pterygopalatine and palatomaxillary sutures are decisive for the quality (parallel or V‑shaped) of the median palatine suture expansion and thus also for the morphological changes of the maxillary palatal vault.

## Age-related changes in maxillary sutures and different RME effects on palatal morphology

The effects of skeletal expansion with RME appliances are largely determined by the point of force application and its relation to the maxillary rotation centres. According to various studies, these are located either in the dorsal part of the median palatine suture or close to the frontomaxillary sutures [2, 6, 15, 38, 44, 45]. However, the marked effects of ageing on palatal morphology suggest that both the position of the centre of resistance and the centre of rotation of the maxilla are not fixed, but change. Melsen and Melsen [29] have already suggested that the centre of resistance of the maxillary complex changes during postnatal development, showing a shift towards the fused sutural region from the juvenile to the pubertal period.

In younger patients up to age 10 (PG1), an almost parallel median palatal suture opening occurs, accompanied by and corresponding to morphological changes in the palatal vault, particularly in the ventral part, and around the palatine processes of the maxilla. There seems to be a mutual interaction between the median and the transverse palatal sutures: The younger the patients are, the more the median palatal suture opens, this influences the transverse palatine suture, which also opens.

Lione et al. [26] interpreted CT data and demonstrated that in younger patients RME treatment significantly increases the distance between the lateral pterygoid processes. However, if the palatomaxillary and the pterygopalatine sutures are also morphologically altered due to age as described in the studies by Melsen and Melsen [29] and Tschechne [40], the bones involved behave as one unit under the therapeutically applied force systems [39]. As a result, the opening of the median palatal suture is met with increased resistance in the posterior region with increasing age [13, 43]. The maxillary centre of resistance is shifted dorsocranially [5] and the centre of rotation is shifted ventrally, resulting in a V-shaped opening of the median palatal suture and a corresponding influence on the morphology of the palatal vault (PG3). As the sphenoidal bone is not paired, the pterygoid process is bent laterally [44] and tensions are created around the sphenoidal bone [17, 20].

The interaction of the various centres of rotation is the cause of palatal height and shape changes after RME treatment. Especially the centres of rotation in the frontal plane near the frontomaxillary sutures cause the bone of the hard palate to rotate and pivot laterally, consequently leading to a relative paramedial increase in palatal height. In younger patients (PG 1), this effect occurs not only in the palatal processes of the maxilla but also in the horizontal plate of the palatine bone. With increasing age and cascading obliterations of the transverse pterygopalatine and palatomaxillary sutures, only the osseous maxillary parts are affected to a significant extent.

## Conclusion

The present study was the first to evaluate the effects of rapid maxillary expansion on the morphology of the maxillary palate in relation to patient age. The changes were analysed in the three spatial planes on dental casts.

The palatal width increased significantly in all cases at all measurement points. While the early treatment group shows a greater width increase posteriorly than anteriorly, this effect is reversed the two later treatment groups. The widening is then significantly greater anteriorly than posteriorly on all levels. This is also confirmed by the results of the width ratio calculation.

The palatal height increases significantly during treatment anteriorly in all three groups, but in the posterior region median and right and left paramedian only in the youngest patient group. With increasing age, the height changes in the posterior region become smaller, resulting in different alterations of palatal depth. Overall, the treatment-induced changes are clearly more pronounced in width than in height.

A parallel and more even opening of the suture from the palate to the nasal floor is age-dependent and happens only in patients with an early treatment begin, whereas a later treatment start leads to a V-shaped opening with a decrease in widening. Treatment success thus significantly depends upon the age at treatment begin.

Visual and metric analyses of CBCT datasets allow the interpretation that age-dependent obliteration tendencies of the sutures have a decisive influence on the biomechanics of the forced skeletal expansion of the maxilla. Of the surrounding structures, in addition to the pterygopalatomaxillary sutures, the transverse palatine suture has a key role for the different morphological effects. Its age-progressive obliteration causes a craniodorsal shift of the maxillary centre of resistance and a ventral shift of the centre of rotation, which is responsible for an altered suture expansion mode while using the same force application.

The partially possible comparison of selected measurement on dental casts and CBCT scans indicates that conclusions about the type of suture opening can be drawn from cast measurements alone. For this indication, radiation- and cost-intensive CBCT datasets appear to be dispensable.

## References

[1] Atac AAT, Karasu HA, Aytac D (2006). Surgically assisted rapid maxillary expansion compared with orthopedic rapid maxillary expansion. Angle Orthod.

[2] Baldini A, Nota A, Santariello C, Caruso S, Assi V, Ballanti F, Gatto R, Cozza P (2018). Sagittal dentoskeletal modifications associated with different activation protocols of rapid maxillary expansion. Eur J Paediatr Dent.

[3] Ballanti F, Lione R, Baccetti T, Franchi L, Cozza P (2010). Treatment and posttreatment skeletal effects of rapid maxillary expansion investigated with low-dose computed tomography in growing subjects. Am J Orthod Dentofacial Orthop.

[4] Bazargani F, Feldmann I, Bondemark L (2013). Three-dimensional analysis of effects of rapid maxillary expansion on facial sutures and bones: a systematic review. Angle Orthod.

[5] Braun S, Bottrel JA, Lee KG, Lunazzi JJ, Legan HL (2000). The biomechanics of rapid maxillary sutural expansion. Am J Orthod Dentofacial Orthop.

[6] Bucci R, D’Antò V, Rongo R, Valletta R, Martina R, Michelotti A (2016). Dental and skeletal effects of palatal expansion techniques: a systematic review of the current evidence from systematic reviews and meta-analyses. J Oral Rehabil.

[7] Chaconas SJ, Caputo AA (1982). Observation of orthopedic force distribution produced by maxillary orthodontic appliances. Am J Orthod Dentofacial Orthop.

[8] Christie KF, Boucher N, Chung CH (2010). Effects of bonded rapid palatal expansion on the transverse dimensions of the maxilla: a cone-beam computed tomography study. Am J Orthod Dentofacial Orthop.

[9] Dahlberg G (1940). Statistical methods for medical and biological students.

[10] da Silva Filho OG, Lara TS, de Almeida AM, da Silva HC (2005). Evaluation of the midpalatal suture during rapid palatal expansion in children: a CT study. J Clin Pediatr Dent.

[11] Davidovitch M, Efstathiou S, Sarne O, Vardimon AD (2005). Skeletal and dental response to rapid maxillary expansion with 2- versus 4-band appliances. Am J Orthod Dentofacial Orthop.

[12] Garib DG, Henriques JF, Janson G, Freitas MR, Coelho RA (2005). Rapid maxillary expansion—tooth tissue-borne versus tooth-borne expanders: a computed tomography evaluation of dentoskeletal effects. Angle Orthod.

[13] Gautam P, Valiathan A, Adhikari R (2007). Stress and displacement patterns in the craniofacial skeleton with rapid maxillary expansion: a finite element method study. Am J Orthod Dentofacial Orthop.

[14] Ghoneima A, Abdel-Fattah E, Hartsfield J, El-Bedwehi A, Kamel A, Kula K (2011). Effects of rapid maxillary expansion on the cranial and circummaxillary sutures. Am J Orthod Dentofacial Orthop.

[15] Haas AJ (1965). The treatment of maxillary deficiency by opening the midpalatal suture. Angle Orthod.

[16] Habersack K, Karoglan A, Sommer B, Benner KU (2007). High-resolution multislice computerized tomography with multiplanar and 3-dimensional reformation imaging in rapid palatal expansion. Am J Orthod Dentofacial Orthop.

[17] Holberg C (2005). Effects of rapid maxillary expansion on the cranial base—an FEM-Analysis. J Orofac Orthop.

[18] Holberg C, Rudzki-Janson I (2006). Stresses at the cranial base induced by rapid maxillary expansion. Angle Orthod.

[19] Isaacson RJ, Ingram AH (1964). Forces produced by rapid maxillary expansion: II. Forces present during treatment. Angle Orthod.

[20] Jafari A, Shetty KS, Kumar M (2003). Study of stress distribution and displacement of various craniofacial structures following application of transverse orthopedic forces—a three-dimensional FEM study. Angle Orthod.

[21] Jain V, Shyagali TR, Kambalyal P, Rajpara Y, Doshi J (2017). Comparison and evaluation of stresses generated by rapid maxillary expansion and the implant-supported rapid maxillary expansion on the craniofacial structures using finite element method of stress analysis. Prog Orthod.

[22] Kajan ZD, Nasab NK, Eghrari N (2018). Quantitative evaluation of midpalatal suture opening and its relation with zygomaticomaxillary suture status in patients aged 7–25 years using cone beam computed tomography images: in an Iranian population. Contemp Clin Dent.

[23] Kinzinger G, Lisson J, Buschhoff C, Hourfar J, Korbmacher-Steiner H (2022). Impact of rapid maxillary expansion on palatal morphology at different dentition stages. Clin Oral Investig.

[24] Knaup B, Yildizhan F, Wehrbein H (2004). Age-related changes in the midpalatal suture. A histomorphometric study. J Orofac Orthop.

[25] Korbmacher H, Schilling A, Püschel K, Amling M, Kahl-Nieke B (2007). Age-dependent three-dimensional microcomputed tomography analysis of the human midpalatal suture. J Orofac Orthop.

[26] Lione R, Ballanti F, Franchi L, Baccetti T, Cozza P (2008). Treatment and posttreatment skeletal effects of rapid maxillary expansion studied with low-dose computed tomography in growing subjects. Am J Orthod Dentofacial Orthop.

[27] Liu S, Xu T, Zou W (2015). Effects of rapid maxillary expansion on the midpalatal suture: a systematic review. Eur J Orthod.

[28] Melsen B (1975). Palatal growth studied on human autopsy material. Am J Orthod.

[29] Melsen B, Melsen F (1982). The postnatal development of the palatomaxillary region studied on human autopsy material. Am J Orthod.

[30] Möhlhenrich SC, Modabber A, Kamal M, Fritz U, Prescher A, Hölzle F (2016). Three-dimensional effects of pterygomaxillary disconnection during surgically assisted rapid palatal expansion: a cadaveric study. Oral Surg Oral Med Oral Pathol Oral Radiol.

[31] Möhlhenrich SC, Ernst K, Peters F, Kniha K, Chhatwani S, Prescher A, Danesh G, Hölzle F, Modabber A (2021). Immediate dental and skeletal influence of distractor position on surgically assisted rapid palatal expansion with or without pterygomaxillary disjunction. Int J Oral Maxillofac Surg.

[32] Persson M, Thilander B (1977). Palatal suture closure in man from 15 to 35 years of age. Am J Orthod Dentofacial Orthop.

[33] Podesser B, Williams S, Crismani AG, Bantleon HP (2007). Evaluation of the effetcs of rapid maxillary expansion in growing children using computer tomography scanning: a pilot study. Eur J Orthod.

[34] Pont A (1909). Der Zahnindex in der Orthodontie. Z Zahnärztl Orthop.

[35] Priyadarshini J, Mahesh CM, Chandrashekar BS, Sundara A, Arun AV, Reddy VP (2017). Stress and displacement patterns in the craniofacial skeleton with rapid maxillary expansion—a finite element method study. Prog Orthod.

[36] Revelo B, Fishman LS (1994). Maturational evaluation of ossification of the midpalatal suture. Am J Orthod Dentofacial Orthop.

[37] Starnbach H, Bayne D, Cleall J, Subtelny JD (1966). Facioskeletal and dental changes resulting from rapid maxillary expansion. Angle Orthod.

[38] Tausche E, Deeb W, Hansen L, Hietschold V, Harzer W, Schneider M (2009). CT analysis of nasal volume changes after surgically-assisted rapid maxillary expansion. J Orofac Orthop.

[39] Timms DJ (1980). A study of basal movement with rapid maxillary expansion. Am J Orthod.

[40] Tschechne S (2005). Die sagittale Entwicklung des Oberkiefers.

[41] Vacher C, Onolfoc JP, Barbet JP (2010). Is the pterygopalatomaxillary suture (sutura sphenomaxillaris) a growing suture in the fetus?. Surg Radiol Anat.

[42] Wehrbein H, Yildizhan F (2001). The mid-palatal suture in young adults. A radiological-histological investigation. Eur J Orthod.

[43] Weissheimer A, de Menezes LM, Mezomo M, Dias DM, de Lima EM, Rizzatto SM (2011). Immediate effects of rapid maxillary expansion with Haas-type and hyrax-type expanders: a randomized clinical trial. Am J Orthod Dentofacial Orthop.

[44] Wertz R (1970). Skeletal and dental changes accompanying rapid midpalatal suture opening. Am J Orthod.

[45] Wertz R, Dreskin M (1977). Midpalatal suture opening: a normative study. Am J Orthod.

[46] Zimring JF, Isaacson RJ (1965). Forces produced by rapid maxillary expansion. III. Forces present during retention. Angle Orthod.

